# Comparison of the effect of hydroxyl propyl methyl cellulose, pectin, and concentrated raisin juice on gluten‐free bread based on rice and foxtail millet flour

**DOI:** 10.1002/fsn3.3741

**Published:** 2023-10-26

**Authors:** Abolghasem Abdollahzadeh, Mohsen Vazifedoost, Zohreh Didar, Mohammad Hossein Haddadkhodaprast, Mohammad Armin

**Affiliations:** ^1^ Department of Food Science and Technology, Neyshabur Branch Islamic Azad University Neyshabur Iran; ^2^ Department of Food Science and Technology Ferdowsi University of Mashhad Mashhad Iran; ^3^ Department of Agronomy and plant Breeding, Sabzevar Branch Islamic Azad University Sabzevar Iran

**Keywords:** celiac, concentrated raisin juice, millet flour, sensory properties, textural characteristics

## Abstract

The nutritional and technological challenges of gluten‐free (GF) bread have increased the need for its modification due to the growing demand for this product, especially from celiac patients. Therefore, the present study aims at evaluating the influence of hydroxyl propyl methyl cellulose (HPMC) at 1% and 2% levels, pectin at 1.5% and 2.5% levels, and concentrated raisin juice (CRJ) at 3% and 4% levels on the dough rheological properties and quality of GF bread based on rice and millet flour. The GF bread prepared with HPMC and incorporating CRJ had higher water absorption, dough development time, and dough stability. In addition, the firmness of GF bread during 24–72 h after baking in the presence of 1% HPMC with 3% and 4% CRJ followed by 2.5% pectin incorporating 3% and 4% CRJ showed a significant decrease compared to the control sample. Further, the color index of GF bread was improved with the addition of HPMC and pectin and the L* index decreased in all GF breads with CRJ. The highest volume was occupied by bread containing 1% HPMC. The results demonstrated that GF bread could be produced from a mixture of rice and millet flour and its technological quality was improved by using 1% HPMC and 3% CRJ. Therefore, it has the necessary potential for high‐scale production and consumption among members of the society.

## Graphical abstract

Sensory properties of GF bread. Hydroxyl propyl methyl cellulose (HPMC); gluten‐free bread (GF); concentrated Raisin Juice (CRJ).

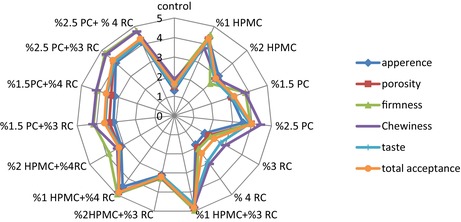

## 1 INTRODUCTION

Today, gastrointestinal diseases are considered as serious health problems, one of which is celiac disease. It is estimated that about 1% of the world population is affected by this disease. Celiac disease is a permanent intolerance to cereal prolamins with a specific oligopeptide sequence by inducing the small intestine inflammation with symptoms of diarrhea, and vomiting (Falcomer et al., 2020; Malalgoda & Simsek, 2017).

This disease can only be treated by completely eliminating gluten from the diet, and these patients should consume gluten‐free (GF) products (Qin et al., 2021). GF breads are diet breads which are safe to use for celiac patients. Various GF flours such as corn, rice, millet, and oak can replace wheat, rye, barley, and oats flours in order to obtain GF breads (Martínez & Gómez, 2017; Qin et al., 2021). Some studies reported that many GF breads produced so far have low nutritional value and do not provide the nutrients required by the human body since most are made with starches and/or refined flours (Thejasri et al., 2017). Rice flour is considered as the most suitable grain flour for producing gluten‐free bread due to minimum levels of prolamin and sodium. In addition, it includes a mild taste and a unique white color. However, GF bread made from rice flour lacks adequate vitamin, mineral, protein, and fiber contents (Qin et al., 2021; Thiranusornkij et al., 2019). Therefore, it is necessary to combine rice flour with other grains, and in the present study foxtail millet (*Setaria italica L*.) was selected to combine with rice flour for producing nutritious GF bread. Millet flour is a rich source of protein (about 12.3 g/100 g), especially essential amino acids, vitamins, minerals (about 3 g/100 g), dietary fiber (14 g/100 g), phytochemicals (phytates, polyphenols, and phytosterols), and micronutrients (such as β carotene) (Chhavi & Sarita, 2012; Thejasri et al., 2017; Tomić et al., 2020).

The factor that creates the appropriate texture in bread is gluten, whose removal and replacement are considered a technological challenge for the production of GF bread (Martínez & Gómez, 2017). Important ingredients are suggested for GF bread such as proteins (Ziobro et al., 2013), enzymes (Yano, 2012), emulsifiers (López‐Tenorio et al., 2015), and hydrocolloids (Hejrani et al., 2021).

Hydrocolloids, as water‐soluble gums, are widely used as an additive in producing bakery products, particularly GF breads. Changing the rheological properties of dough and bakery products to preserve the quality of final products is considered as the functional effects of hydrocolloids (Lazaridou et al., 2007). These compounds are essential for formulating GF breads to create gluten‐like properties and improve the product texture, mouth feel, and appearance (Mariotti et al., 2013; Matos & Rosell, 2015). Additionally, hydrocolloids create a cellular network which stores CO_2_ during fermentation leading to an increase in the volume, texture, and the final quality of bread, as well as improving dough cohesiveness and viscosity through stimulating and attaching starch granules and moisture control (Hejrani et al., 2021; Hu et al., 2020; Kaur et al., 2013).

The hydroxy propyl methyl cellulose (HPMC) as nonionic modified cellulose could be used as a stabilizer and thickener. It has a high potential for creating a hydrocolloid network to replace the gluten in order to prevent the rupture of bread texture, moisture loss, and increase dough consistency, specific volume of bread, and porosity of crumb (Chen et al., 2023). A study has shown that the addition of 1% HPMC leads to an improvement in the internal structure of bread and an increase in the level of porosity in GF bread based on rice and buckwheat flour (Baldino et al., 2018). In previous studies, the effects of HPMC on properties of GF bread from rice flour (Srikanlaya et al., 2022), different starch sources (rice flour or maize starch) (Belorio & Gómez, 2020), and potato starch (Chen et al., 2023) were studied. Pectin is another gum suggested for GF bread in which polysaccharide is derived from citrus peel or apple pomace. Pectin has a high number of hydroxyl groups which can improve the specific volume, crust, and crumb color, as well as texture and elasticity of GF bread. In previous studies, it was suggested that the use of pectin increased the volume and elasticity of GF dough (Jinxin et al., 2018; Sciarini et al., 2012). It has also been reported that the increase in pasting properties is due to the presence of pectin, which has a higher water absorption capacity and thickening power (Bugarín & Gómez, 2023; Ma et al., 2019).

Concentrated raisin juice (CRJ) can be addressed as another additive affecting the nutritional quality and technology of GF breads, which contains trace elements such as Ca, Mg, P, Na, and K, as well as vitamins like A, B, and C (Sabanis et al., 2008). In the CRJ, the sugars are in the form of glucose and fructose, which easily enter the bloodstream without the need for digestion and reduce the hardness of GF bread because of decreasing retrogression. Grapes also have a low glycemic index, meaning that some diabetic diets may be able to safely include them due to their low glycemic load. In addition, grapes contain polyphenols that potentially reduce hyperglycemia, or high blood sugar, which is beneficial for people with diabetes (Batu, 2005; Jankar et al., 2019; Sabanis et al., 2008).

The composition of psyllium husk powder (Fratelli et al., 2021), soybean extruded–expelled meal and pregelatinized cassava starch (Genevois & de Escalada Pla, 2021), sunflower protein concentrate addition on a flour basis mixture (70% rice flour and 30% cornstarch) (Zorzi et al., 2020), quinoa flour (Azizi et al., 2020), and brown rice flour (Luo et al., 2021) have been used to improve the characteristics of GF bread. So far, no study has been published on the quality of the GF bread produced from rice and foxtail millet incorporation with CRJ to the best of our knowledge. Given the benefit of millet flour and CRJ, the present study focused on understanding the properties of the GF bread prepared from rice and millet flour by using hydrocolloids and CRJ.

## 2 MATERIALS AND METHODS

### 2.1 Chemicals

TDF‐100A kit was prepared by Sigma Company (St. Louis, MO). Sulfuric acid, copper (II) sulfate, sodium sulfate, hydrochloric acid, ethylene diamine tetraacetic acid, and boric acid were purchased from Merck Company (Germany).

### 2.2 Raw materials

Commercial millet (*Setaria italica*) and rice flour (Tarom variety) were procured from Zarrin Mill Factory (Mashhad, Iran). Additionally, the HPMC and pectin were obtained from Fluka Co. (Germany) and Silva Co. (Italy), respectively. The compressed bakery yeast (Molasses Co., Iran) and other ingredients (e.g., salt, sugar, and oil) were purchased from local market.

### 2.3 Preparation of CRJ

First, the operations of washing, brewing, and drying of raisins were done, and then using an abrasive mill, small cuts were made for water penetration in its wall. In order to prepare a suitable concentrate from grapes, all the waste materials such as the tail part must be separated first because the tail part lacks nutrients compared to the grape seed itself. The raisin sample was mixed with water (2:1, W/V) and extraction was done at a temperature of 55–77°C under stirring for 4 h. To complete the extraction process, the sample was placed in a water bath at boiling temperature. The extraction process was carried out until the solution brix remained constant (during 2 h). The obtained extract was filtered and concentrated by a rotary evaporator under vacuum (LABOROTA‐40, Heidolph, Germany) at a temperature of 50°C to Brix 70. The sample was put at 50°C for 24 h under 600 rpm in a vacuum rotary evaporator.

### 2.4 GF bread production

Rice and millet flours were used in a ratio of 1:1, and other ingredients including HPMC (1% and 2%), pectin (1.5% and 2.5%), CRJ (3% and 4%), bakery yeast (1%), salt (1%), oil (4%), soy milk powder (2%), and sufficient water (based on the farinograph water absorption level, 49.83%–74.13%, Table 1) were added to the flour mixture. The dough was mixed using a dough mixer (EB124101) at 40 rpm for 15 min. The dough was initially fermented at 30°C for 30 min under a relative humidity of 75%. Then, it was divided into 400‐g pieces, rounded, and placed at 30°C for 10 min for the intermediate fermentation. The dough was proofed at 35°C and 90% moisture for 60 min, followed by baking in a convection multilayer industrial oven (Karl welker kg, Germany) equipped with indirect thermal circulation system and steam generator system to supply steam to the layers at 210°C for 30 min. After cooling at room temperature for 1 h, the bread was packed and sealed in polyethylene bags, and stored at a temperature of 20°C in a relative humidity of 50% until further use.

**TABLE 1:** Water absorption of treatments.

Treatment	Water absorption (%)
Control	49.83^e^ ± 0.35
1% HPMC	62.83^c^ ± 0.66
2% HPMC	73.6^a^ ± 0.75
1.5% pectin	56.13^d^ ± 0.8
2.5% pectin	67.53^b^ ± 0.45
3% CRJ	50.20^e^ ± 0.30
4% CRJ	50.23^e^ ± 0.3
1% HPMC + 3% CRJ	62.66^c^ ± 0.45
1% HPMC + 4% CRJ	63.36^c^ ± 0.65
2% HPMC + 3% CRJ	74.13^a^ ± 0.3
2% HPMC + 4% CRJ	74.06^a^ ± 0.25
1.5% pectin + 3% CRJ	56.23^d^ ± 0.75
1.5% pectin + 4% CRJ	56.46^d^ ± 0.5
2.5% pectin + 3% CRJ	67.86^b^ ± 0.65
2.5% pectin + 4% CRJ	68.10^b^ ± 0.70

*Note*: Means followed by different letters in a column are significantly different at *p* < .05.

### 2.5 Chemical analysis of flour

Millet and rice flours were tested for chemical properties such as moisture, ash, protein, fat, fiber, and pH based on the AACC standard (2000). Moisture content was determined by drying method at 105 ± 1°C until constant weight. Ash content was measured by burning of samples at 550°C using a muffle furnace for 8 h. Protein was determined as total nitrogen by Kjeldahl method. A correction factor of 6.25 was used to convert the amount of nitrogen into protein. Fat content was obtained according to the International Organization for Standardization using the Soxhlet method. The total, soluble, and insoluble dietary fiber were analyzed by an enzymatic–gravimetric method using the TDF‐100A. To measure the pH, a sample of grated bread (10 g) were uniformly mixed with distilled water (15 mL) and then the pH was measured using a pH meter (American Association of Cereal Chemists, 2000).

### 2.6 Farinograph test

The farinograph is often used to calculate water absorption (%), dough development time (min), dough stability (min), and degree of softening (B.U) in GF breads, as there is no evidence that a single rheology guarantees an optimum in three times. The farinograph test was performed using a Farinograph device (Brabender, Germany) following AACC 54‐21 method.

### 2.7 Physicochemical characteristics of the GF bread

Moisture content of the GF bread was determined according to Jinxin et al. (2018) procedure. First, the bread samples were weighed and then placed in a 130°C oven. After 2 h, the samples were placed in a desiccator and after reaching a constant weight, their weight were measured. Based on the weight difference before and after placing in the oven, the moisture percentage of the samples was measured. Further, the loaf volume was measured through rapeseed displacement method based on the AACC 10‐72 (2000), and specific volume was calculated as volume/weight (cm^3^/g). Regarding the porosity of the bread, a scanner (HP Scanjet G3010, Merican) was utilized to photograph the 2 × 2 cm pieces of the bread crumb, the images of which were saved as JPG. Then, the images were analyzed by using image processing and analysis in Java software (Image J), in which the part bit was activated, gray level was created, and the ratio of bright to dark points was defined as a porosity percentage. All experiments were carried out three times (Hejrani et al., 2017).

### 2.8 Color measurement

The 2 × 2 cm slices of bread were imaged using a scanner (HP Scanjet G3010; resolution = 300 p) and examined by activating the LAB space in the plugin part. The L* index represents the brightness and ranges from 0 to 100. Furthermore, a* indicates a significant amount of color close to green and red, while b* refers to a major level of color close to blue and yellow color. All experiments were carried out three times (Hejrani et al., 2019).

### 2.9 Texture analysis

The firmness of GF bread (20 mm thickness) and change in its texture due to staling were determined using the penetration test. A QTS texture analyzer (CNS Farnell, UK) was used to measure the force required to penetrate a round‐bottom probe (2 cm diameter, 2 cm height, and 25 mm thickness) at a velocity of 10 mm/min and descend it up to 24 mm. Three replicates from bread stored during different amounts of time (24, 48, and 72 h) at ambient temperature after baking were analyzed (Majzoobi et al., 2015).

### 2.10 Sensory analysis

A hedonic sensory evaluation of the breads (i.e., odor, taste, flavor, upper surface properties, firmness, porosity, and total acceptance) was conducted with 70 untrained volunteers (between 18 and 66 years old, containing 35 men and 35 women). Samples were analyzed three times after baking and cooling at room temperature for 1 h and presented in slices (20 mm thick cut in disposable plastic plates). The panelists have been requested not to use cigarettes and spicy food before the measurement and to drink water after consuming each sample. The samples were coded based on the number and the measurement was done in a room with proper ventilation and sufficient light. On this scale, score ranges from 1 (least pleasure) to 5 (best pleasure) (Pertuzatti et al., 2015).

### 2.11 Statistical analysis

Statistical analyses were performed with SPSS software (version 19). The mean of the data was analyzed by one‐way analysis of variance (ANOVA) in terms of significance, and the difference between them was evaluated at the 95% level using Duncan's test. Chemical analysis, farinograph test, physicochemical characteristics, color evaluation, texture analysis, and sensory analysis of bread samples were statistically analyzed as dependent variables.

## 3 RESULTS AND DISCUSSION

### 3.1 Chemical analysis of flour

Table 2 summarizes the chemical composition of rice and millet flour. The results revealed higher amounts of ash (1.95% ± 0.5%), protein (11.03% ± 0.5%), cured fat (2.05% ± 0.3%), and fiber (5.8% ± 0.3%) in millet flour compared to the rice one. However, the pH of rice flour (6.28 ± 0.2) was greater than that of the millet flour (5.87 ± 0.3). The moisture content of millet and rice flour was estimated at 8.12% ± 0.3% and 8.39% ± 0.2%, respectively, which were not significantly different (*p* > .05). The millet proteins can improve the quality of GF bread (Storck et al., 2013; Taghdir et al., 2017). Further, fat and ash contents usually indicate the levels of energy and minerals in flour, respectively. In the present study, millet flour had more fat and ash than the rice one, which is consistent with the results of Emire and Tiruneh (2012), Tomić et al. (2020), and Sarabhai et al. (2021) which demonstrated a higher amount of protein, fiber, and ash in the flour prepared from millet (Emire & Tiruneh, 2012; Sarabhai et al., 2021; Tomić et al., 2020).

**TABLE 2:** Chemical compositions of rice flour and millet flour.

Treatment	Moisture (%)	Ash (%)	Protein (%)	Fiber (%)	Crude fat (%)	pH
Rice flour	8.39^a^ ± 0.2	0.89^b^ ± 0.1	8.25^b^ ± 0.4	0.45^b^ ± 0.1	1.19^b^ ± 0.3	6.28^a^ ± 0.2
Millet flour	8.12^a^ ± 0.3	1.95^a^ ± 0.2	11.03^a^ ± 0.5	5.8^a^ ± 0.3	2.05^a^ ± 0.5	5.87^b^ ± 0.3

*Note*: Means followed by different letters in a column are significantly different at *p* < .05.

### 3.2 Dough properties

Based on Table 1, The water absorption percentage of the samples ranged from 49.83% ± 0.35% (control sample) to 74.13% ± 0.3% (2% HPMC + 3% CRJ). In general, adding separate HPMC and pectin and increasing their concentration caused a significant increase in the water absorption of the samples, but adding CRJ in both concentrations did not cause a significant change in the water absorption of the samples compared to the control. The water absorption was maximized in the GF breads containing 2% HPMC + 3% CRJ, 2% HPMC + 4% CRJ, and 2% HPMC with no significant difference (*p* > .05), while the minimum was related to the control, 3% CRJ, and 4% CRJ. Thus, the HPMC (62.83%–73.6%) was more effective on water absorption in comparison with pectin (56.13%–67.53%). Compared to the control, the CRJ influenced water absorption in combination with HPMC although its effect was not more than that of pectin and HPMC. However, no significant difference was observed between the used concentrations of CRJ (3% and 4%). The CRJ is composed of sugars (e.g., glucose and fructose) and fiber which retain and hold moisture. Table 3 outlines the farinograph parameters of dough like dough development time (DDT), stability, and degree of softening (Brabender Unit, B.U) under the effect of pectin, HPMC, and CRJ concentrations. Given the lower consistency of gluten‐free flour than the wheat one, 230 BU line was considered as a standard for farinograph tests in the dough prepared with rice and millet flour (Cappa et al., 2016). The time necessary to reach a dough consistency (dough development time, DDT) of 230 BU changed in the samples produced with pectin and HPMC. The DDT was significantly more in the sample consisting of 2% HPMC + 4% in following 2% HPMC, and 2% HPMC + 3% CRJ (no significant difference) than the GF bread produced with pectin and control. Also, the lowest DDT was obtained in control samples and 3% and 4% of CRJ, which are not significantly different from each other (*p* > .05). This effect can be attributed to the high ability of HPMC to absorb more water through hydrogen bonding, leading to greater DDT. The flour strength is represented by stability value, and higher levels indicate stronger dough. Additionally, the use of pectin, HPMC, and CRJ affected stability. The stability value reflected dough strength, and the higher and lower values were obtained in the samples of 2% HPMC (8.36 min), and control (2.5 min), respectively. The 2% HPMC + 3% CRJ (26.56 B.U) and the control (59.20 B.U) samples recorded the lowest and highest degree of softening (mixing tolerance index), respectively, which indicates the effect of HPMC and CRJ together in softening the bread. In general, CRJ in combination with HPMC had a significant effect in improving the properties of bread, which is probably due to the interaction between the compounds of these two substances and the formation of new bonds. Also, HPMC has been more effective than pectin in producing high‐quality GF bread. The results of farinograph tests are in line with those of other studies (Shalini & Laxmi, 2007). The greater stability of the dough after the addition of hydrocolloids can be explained by the formation of a three‐dimensional network (e.g., gluten one) that strengthens the dough. As a result, improving the dough will strengthen the characteristics of the bread (Lazaridou et al., 2007). The negative effect of pectin on the farinographic characteristics of Chinese steamed bread has been reported, which is due to increased water absorption and decreased DDT and dough stability time (Jinxin et al., 2018).

**TABLE 3:** The rheological parameters of dough affected HPMC, pectin, and CRJ.

Treatment	Dough development time (min)	Dough stability (min)	Degree of softening (B.U)
Control	3.33^f^ ± 0.1	2.50^f^ ± 0.1	59.20^a^ ± 0.35
1% HPMC	4.80^d^ ± 0.15	4.63^c^ ± 0.1	40.56^d^ ± 0.45
2% HPMC	7.8^b^ ± 0.1	8.36^a^ ± 0.15	28.1^f^ ± 0.09
1.5% pectin	4.56^e^ ± 0.15	3.60^d^ ± 0.10	48.53^b^ ± 0.20
2.5% pectin	6.93^c^ ± 0.15	7.40^b^ ± 0.4	33.86^e^ ± 0.35
3% CRJ	3.50^f^ ± 0.20	2.56^e^ ± 0.15	58.63^a^ ± 0.9
4% CRJ	3.43^f^ ± 0.05	2.66^e^ ± 0.2	58.83^a^ ± 0.6
1% HPMC + 3% CRJ	4.90^d^ ± 0.10	4.53^c^ ± 0.30	40.46^d^ ± 0.70
1% HPMC + 4% CRJ	5^d^ ± 0.10	4.53^c^ ± 0.35	41.66^d^ ± 1.5
2% HPMC + 3% CRJ	7.8^b^ ± 0.10	8.2^a^ ± 0.10	26.56^g^ ± 0.5
2% HPMC + 4% CRJ	7.97^a^ ± 0.05	8.13^a^ ± 0.2	28.03^f^ ± 0.9
1.5% pectin + 3% CRJ	4.60^de^ ± 0.25	3.6^d^ ± 0.17	48.36^b^ ± 0.30
1.5% pectin + 4% CRJ	4.46^e^ ± 0.1	3.73^d^ ± 0.2	47.76^c^ ± 0.3
2.5% pectin + 3% CRJ	6.83^c^ ± 0.1	7.20^b^ ± 0.10	33.86^e^ ± 0.20
2.5% pectin + 4% CRJ	6.93^c^ ± 0.15	7.33^b^ ± 0.1	33.43^e^ ± 0.3

*Note*: Means followed by different letters in a column are significantly different at *p* < .05.

### 3.3 Moisture content of the GF bread prepared with HPMC, pectin, and CRJ

Table 4 presents the effect of pectin, HPMC, and CRJ on the moisture content of the GF bread produced from millet and rice flour. The results revealed that the control and GF bread with various formulations were significantly different with respect to moisture content (*p* < .05). The level of the parameter varied in the range of 17.06% ± 0.1%–28.45% ± 0.45%, and all samples had significantly more moisture than the control. As shown in Table 4, the moisture content of GF bread significantly increases following the application of pectin, HPMC, and CRJ. The highest amount of moisture was observed in the GF bread containing 2% HPMC + 4% CRJ (*p* < .05). However, the parameter was minimized in the control bread based on rice and foxtail millet flour without additives. The difference in moisture content can be ascribed to the water‐holding capacity of the HPMC, pectin, and CRJ. Further, the GF bread based on rice and foxtail millet diminished moisture, suggesting that the lack of gluten decreased the moisture maintenance in the crumb, and addition of gums compensated the absence of gluten. The HPMC was more effective on moisture than the pectin and CRJ. Therefore, HPMC could well improve water retention in the crumb due to its desirable properties in elevating water absorption. The results of some studies have confirmed the effect of HPMC gum on improved GF bread (Encina‐Zelada et al., 2019; Sabanis & Tzia, 2011).

**TABLE 4:** The physicochemical parameters of GF bread affected HPMC, pectin, and CRJ.

Treatment	Specific volume (cc/g)	Porosity (%)	Moisture content (%)
Control	2.55^m^ ± 0.00	19.85^j^ ± 0.26	17.16^k^ ± 0.45
1% HPMC	4.05^e^ ± 0.03	37.10^b^ ± 0.51	21.15^h^ ± 0.10
2% HPMC	2.92^l^ ± 0.01	33.55^e^ ± 0.42	24.57^de^ ± 0.11
1.5% pectin	3.17^i^ ± 0.01	31.46^g^ ± 0.81	20.96^h^ ± 0.16
2.5% pectin	3.80^f^ ± 0.01	35.3^c^ ± 0.37	23.60^f^ ± 0.42
3% CRJ	3.2^k^ ± 0.01	25.52^i^ ± 0.20	18.50^j^ ± 0.20
4% CRJ	3.22^i^ ± 0.01	26.83^h^ ± 0.74	19.23^i^ ± 0.32
1% HPMC + 3% CRJ	4.49^b^ ± 0.02	39.10^a^ ± 0.52	23.85^f^ ± 0.22
1% HPMC + 4% CRJ	4.58^a^ ± 0.01	39.88^a^ ± 0.49	24.28^e^ ± 0.19
2% HPMC + 3% CRJ	3.06^j^ ± 0.00	34.79^d^ ± 0.11	27.27^b^ ± 0.11
2% HPMC + 4% CRJ	3.08^i^ ± 0.01	35.06^c^ ± 0.10	28.45^a^ ± 0.10
1.5% pectin + 3% CRJ	3.51^h^ ± 0.01	32.87^f^ ± 0.16	22.34^g^ ± 0.16
1.5% pectin + 4% CRJ	3.66^g^ ± 0.00	33.95^e^ ± 0.26	22.97^g^ ± 0.26
2.5% pectin + 3% CRJ	4.21^d^ ± 0.00	37.11^b^ ± 0.42	24.90^d^ ± 0.42
2.5% pectin + 4% CRJ	4.34^c^ ± 0.00	37.87^b^ ± 0.55	25.16^c^ ± 0.15

*Note*: Means followed by different letters in a column are significantly different at *p* < .05.

### 3.4 Specific volume and porosity of GF bread properties

Since the specific volume and porosity demonstrate the gas‐holding capability of the bread, they are considered as key factors for consumer acceptance (Hejrani et al., 2021). The incorporation of gums and CRJ led to a significant increase in the specific volume of GF bread compared to the application of gums alone (Table 5). Among all the treated and control GF breads, the sample of 1% HPMC + 4% CRJ (*p* > .05) exhibited higher specific volume, followed by those with 1% HPMC + 3% CRJ, and 2.5% pectin + 3% and 4% CRJ (*p* > .05). However, a lower volume was found in the control since dough could not hold gas during proofing due to the lack of gluten. The variation in specific volume was mainly influenced by the additives added to the GF bread prepared from rice and foxtail millet. Regarding porosity, the same trend was observed so that the parameter was maximized in the GF bread containing 1% HPMC + 3% and 4% CRJ (39.10% ± 0.52% and 39.88% ± 0.49%, respectively) compared to the control (19.85% ± 0.26%).

**TABLE 5:** The color analysis of GF bread affected HPMC, pectin, and CRJ.

Treatment	L*	a*	b*
Control	36.18^i^ ± 0.31	3.25^d^ ± 0.04	14.3^bc^ ± 0.1
1% HPMC	48.18^a^ ± 0.12	3.08^e^ ± 0.06	14.53^b^ ± 0.15
2% HPMC	43.42^d^ ± 0.25	3.10^e^ ± 0.01	14.66^b^ ± 0.05
1.5% pectin	44.04^c^ ± 0.36	3.35^c^ ± 0.02	14.73^ab^ ± 0.15
2.5% pectin	45.41^b^ ± 0.25	3.32^c^ ± 0.02	14.87^a^ ± 0.05
3% CRJ	29.88^m^ ± 0.07	3.39^bc^ ± 0.14	14.23^c^ ± 0.05
4% CRJ	25.45^n^ ± 0.24	3.8^a^ ± 0.19	14.1^d^ ± 0.1
1% HPMC + 3% CRJ	42.82^e^ ± 0.1	3.06^f^ ± 0.08	14.20^cd^ ± 0.1
1% HPMC + 4% CRJ	35.24^j^ ± 0.25	3.32^d^ ± 0.23	14.03^d^ ± 0.05
2% HPMC + 3% CRJ	38.38^g^ ± 0.24	3.33^c^ ± 0.14	14.40^b^ ± 0.15
2% HPMC + 4% CRJ	34.57^k^ ± 0.45	3.34^c^ ± 0.06	14.40^b^ ± 0.1
1.5% pectin + 3% CRJ	37.76^h^ ± 0.24	3.55^b^ ± 0.08	14.63^b^ ± 0.05
1.5% pectin + 4% CRJ	30.29^l^ ± 0.23	3.57^b^ ± 0.08	14.13^d^ ± 0.5
2.5% pectin + 3% CRJ	40.71^f^ ± 0.52	3.55^b^ ± 0.01	14.63^b^ ± 0.5
2.5% pectin + 4% CRJ	34.3^i^ ± 0.5	3.35^c^ ± 0.02	14.30^bc^ ± 0.1

*Note*: Means followed by different letters in a column are significantly different at *p* < .05.

The results indicated a decline in the porosity and specific volume by promoting the concentration of HPMC from 1% to 2%. Furthermore, the effect of HPMC incorporating 3% and 4% CRJ on the improvement of porosity and specific volume parameters was more than that of pectin and CRJ with 3% and 4% concentration. Accordingly, the highest specific volume was obtained by combining the appropriate levels of gum and CRJ. A rise in the concentration of HPMC decreased specific volume due to more water binding in dough, causing the reduced dough consistency, large hole formation, and plastic structure in the GF bread (Mancebo et al., 2017; Sabanis & Tzia, 2011). As already mentioned, the CRJ contains tartaric acid, which affects fermentation action. It consists of fermentable sugars (glucose and fructose), which produce more gases with the yeast during fermentation (Torley & Van der Molen, 2005). In addition, the raisin acts as a humectant, which improves dough development and bread volume (Sabanis et al., 2009; Sheikholeslami et al., 2018). A rise in the concentration of HPMC decreased specific volume due to more water binding in dough, causing the reduced dough consistency, large hole formation, and plastic structure in the GF bread (Mancebo et al., 2017; Sabanis & Tzia, 2011). As already mentioned, the CRJ contains tartaric acid, which affects fermentation action. It consists of fermentable sugars (glucose and fructose), which produce more gases with the yeast during fermentation (Torley & Van der Molen, 2005). In addition, the raisin acts as a humectant, which improves dough development and bread volume (Sabanis et al., 2009; Sheikholeslami et al., 2018).

The hydrocolloid addition to formulation enhanced the viscosity due to the nature of hydroxyl group in gums. Thus, more air bubbles were kept in dough during proofing and baking, and the large number of cell gases increased the specific volume of bread. The results of many studies have confirmed that HPMC maintains the homogeneity of the system, improves the viscosity, and strengthens gas cell walls, leading to more gas retention, and consequently greater volume and porosity in GF bread (Morreale et al., 2018; Sahagún & Gómez, 2018). HPMC hydrocolloid increased the final specific volume of bread more than psyllium and xanthan gums (Belorio & Gómez, 2020). The highest specific volume has already been found when using HPMC, comparing breads prepared with corn starch or rice flour (Mancebo et al., 2015; Martínez & Gómez, 2017). A higher specific volume has been reported in GF bread texture containing HPMC compared to propylene glycol alginate gum (Zhao et al., 2021). Martínez and Gómez (2017) attributed the higher consistency of rice flour dough to corn starch, which is probably due to the presence of protein in rice flour (Martínez & Gómez, 2017) which indicates the potential of rice flour protein to form dough suitable for bread production compared to other grains. The behavior of HPMC is related to its capacity to form a thermally reversible gel during curing, which increases viscosity and creates gas cell walls, providing high volume by preventing moisture loss (Belorio & Gómez, 2020).

### 3.5 Firmness of GF bread

**FIGURE 1:**
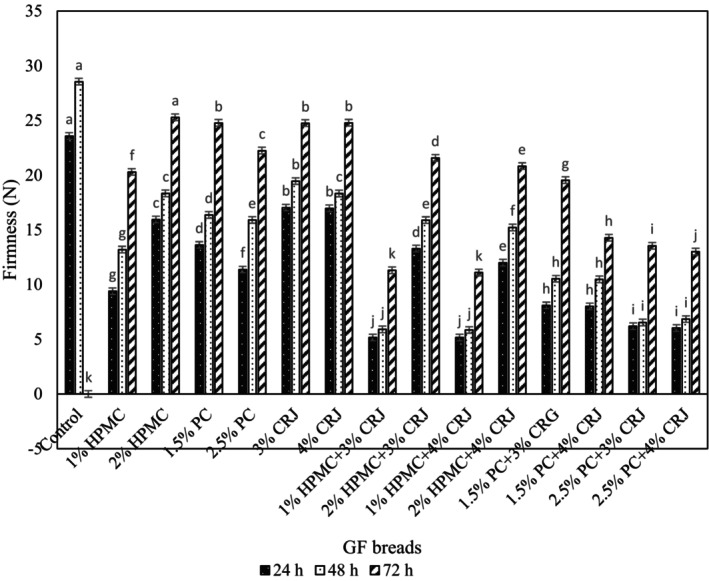
The firmness of GF bread during storage.

The GF bread texture, especially the firmness of crumb, is considered as a degree of staleness. The firmness of crumb reflects its resistance to shape changes during storage time, and is a degree of bread staleness (Martínez & Gómez, 2017). Figure 1 displays the results of the firmness of the GF bread during storage (24–72 h). The HPMC and pectin incorporation with CRJ have a positive effect on GF bread. The addition of both pectin and HPMC individually was more effective in decreasing bread firmness and delaying the staling than the CRJ at 3% and 4%. However, the breads prepared with pectin and HPMC incorporating CRJ exhibited the minimum firmness and staling during the storage among all the GF bread. Also, control bread has maximum firmness during 72 h. The treatment of 1% HPMC + 3% CRJ and 1% HPMC + 4% CRJ (without significant difference, *p* > .05) diminished crumb firmness due to the water‐binding capacity of HPMC through hydrogen bonding and avoided water loss during storage to 72 h. Furthermore, the crumb firmness of the samples prepared by adding 2% HPMC alone and in combination with CRJ at different levels was more than that of those with 1% HPMC. In a previous study, it was reported that the further concentration of HPMC increases water absorption, leading to lower volume and denser crumb, and found a higher crumb firmness due to the inverse relationship between volume and firmness (Mancebo et al., 2017). In the present study, increasing the concentration of pectin from 1.5% to 2.5% caused a decrease in the hardness of the sample. Compared to the pectin gums, 1% HPMC gum was more effective in reducing firmness, which can be explained by its higher water retention ability, as well as keeping more moisture in crumb during storage to 72 h, minimizing the staling of GF bread. According to the obtained results, in the samples of 1% HPMC + 3% CRJ, 1% HPMC + 4% CRJ, 2% HPMC + 4% CRJ, 1.5% pectin + 3% CRJ, 1.5% pectin + 4% CRJ, 2.5% pectin + 3% CRJ, and 2.5% pectin + 4% CRJ, there is no significant difference in hardness between 24 and 48 h after baking (*p* < .05), but there is a significant difference with 72 h postbaking (*p* > .05), explaining the slower staleness in the samples. At 24 h after baking, the firmness of the control sample was 23.57 N, which elevated to 28.53 N after 48 h baking. The parameter could not be measured at 72 h due to the lack of suitable tissue. Based on the results, adding an appropriate concentration of HPMC incorporating CRJ improved the staling of the GF bread because sorbitol of CRJ as a humectant helps to keep water in crumb during storage, delaying the staling (Sabanis et al., 2008; Soukoulis & Tzia, 2018).

In line with the results of our study, the minimum firmness of GF bread after using the optimal level of HPMC, as well as the reduction of staleness of GF bread during storage was confirmed (Sabanis & Tzia, 2011; Sivaramakrishnan et al., 2004). A study suggested that pectin at a proper concentration enhances textural properties and decreases the firmness of GF bread (Jinxin et al., 2018).

### 3.6 Color of GF bread crust

Color is an important feature in the sensory evaluation of food products that affect their acceptance or rejection and L*, a*, and b* parameters are international color measurement standards. As shown in Table 5, the brightness (L*) of the GF bread based on the foxtail millet and rice flour varies between 25.44 ± 0.24 in the sample containing 4% CRJ and 48.18 ± 0.12 in that with 1% HPMC. The highest brightness is obtained in the GF bread containing 1% HPMC, followed by the samples of 2.5% pectin, 1.5% pectin, and 2% HPMC with significant differences (*p* < .05). Hydrocolloid presents water‐holding capacity, which prevents water loss from crumb to crust, and subsequently diminishes the changes in bread crust and reduces surface shrinkage, which influences an increase in the L* crust (Mandala et al., 2008). Morreale et al. (2018) reported greater lightness and color index following the use of HPMC in the GF bread based on rice flour (Morreale et al., 2018).

The results demonstrated the darker crust color (small values of L*) in the GF breads prepared with CRJ (3% and 4%) and those treated with pectin and HPMC incorporating CRJ compared to the others. In terms of a* and b*, small significant differences were found among the samples treated and no clear tendency was observed (*p* < .05). The a* factor of the 4% CRG sample is higher than the other samples, which indicates the greater redness of the sample, followed by the 2.5% pectin with 3% and 4% CRJ, the 1.5% pectin sample with 3% and 4% CRJ, and 3% CRJ. Samples containing 2.5% pectin and 1% HPMC + 4% CRJ had the highest and lowest b* values, respectively.

In the GF bread containing CRJ, the low levels of L* and high level of a* can be attributed to the presence of high amounts of reducing sugars, which participate in color reactions (caramelization and Maillard) and darkens the color of the bread (Sabanis et al., 2008). The Maillard reaction between amino acids and reducing sugars, as well as sugar caramelization, are responsible for changing the color of the bread crust (Purlis & Salvadori, 2009). Also, water activity can vary depending on the hydrocolloid and hydration of the pastes, and this can cause Maillard reactions that contribute to the mobility of the reactants (Gonzales et al., 2010). Many studies reported the addition of concentrated raisin into bread improves the golden brown color of crust due to decreased brightness and more color intensity (Sabanis et al., 2008; Soukoulis & Tzia, 2018).

### 3.7 Sensory properties

**FIGURE 2:**
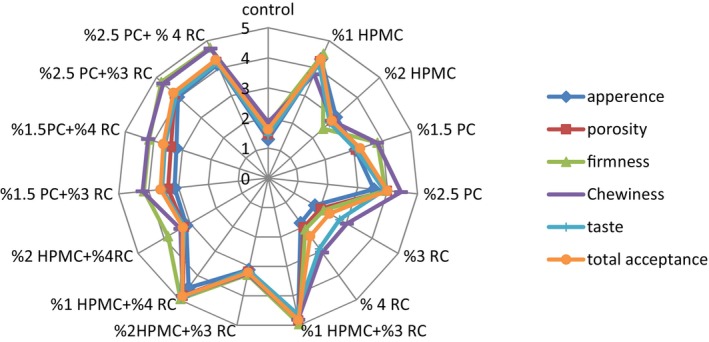
Sensory properties of GF bread.

The sensory properties of the GF bread prepared with HPMC, pectin, and CRJ, as well as an incorporation of gums and CRJ were evaluated by untrained panelists. The score of sensory characteristics was maximized in the GF bread prepared with 1% HPMC + 3% and 4% CRJ and 2.5% pectin + 3% and 4% CRJ without any significant difference, respectively. However, the lowest score was related to the control sample based on the foxtail millet and rice flour (*p* < .05) (Figure 2). The results of the comparison between the treatment with HPMC and pectin revealed that the bread with 1% HPMC had the highest score of all sensory properties, followed by the sample with 2.5% pectin. Further, the incorporation of HPMC and pectin with CRJ at both 3% and 4% levels without any significant difference (*p* > .05) in the GF bread significantly increased sensory characteristics (*p* < .05) and it was more preferable by the panelists. The results indicated the significant effect of applying pectin and HPMC with CRJ on the sensory attributes (e.g., firmness, appearance, porosity, chewiness, taste, and total acceptance) of GF bread (*p* < .05). In accordance with the results of this study, it has been suggested in different studies to increase the sensory characteristics of GF bread by using hydrocolloid in their fermentation (Encina‐Zelada et al., 2019; Kaur et al., 2015; Morreale et al., 2018). Furthermore, Sheikholeslami et al. (2018) and Sabanis and Tzia (2011) emphasized the better sensory properties of bread after adding concentrated raisin in its formulation (Sabanis & Tzia, 2011; Sheikholeslami et al., 2018).

## 4 CONCLUSIONS

The addition of HPMC and pectin alone and in combination with CRJ to the GF bread formulation could improve the properties measured by the equipment, specific volume, porosity, staling, sensory properties, and overall quality of the GF bread based on rice and foxtail millet. In addition, the HPMC at 1% level was more effective in improving the quality of bread, even more than pectin. The incorporation of 1% HPMC with CRJ at 3% and 4% levels led to the best result in terms of all the parameters under study although 3% CRJ was selected due to economic issues. The results of the present study can provide better insights into the interactions between hydrocolloid and CRJ to improve the characteristics of the GF bread based on the rice and foxtail millet.

## AUTHOR CONTRIBUTIONS

**Abolghasem Abdollahzadeh:** Data curation (equal); formal analysis (equal); investigation (equal); methodology (equal); writing – original draft (equal). **Mohsen Vazifedoost:** Formal analysis (equal); investigation (equal); project administration (equal); writing – original draft (equal). **Zohreh Didar:** Investigation (equal); methodology (equal); resources (equal). **Mohammad Hossein Haddadkhodaprast:** Validation (equal); writing – review and editing (equal). **Mohammad Armin:** Software (equal); validation (equal).

## FUNDING INFORMATION

This work was funded by the Department of Food Science &Technology, Sabzevar Branch, Sabzevar Bread Researchers Food Industry Company located in Sabzevar Industrial Town, and also Neyshabur Branch, Islamic Azad University.

## CONFLICT OF INTEREST STATEMENT

The authors have declared no conflict of interest.

## ETHICS STATEMENT

This article does not contain any studies with human or animal subjects.

## Data Availability

Research data are not shared.
